# Gateway-Assisted Vector Construction to Facilitate Expression of Foreign Proteins in the Chloroplast of Single Celled Algae

**DOI:** 10.1371/journal.pone.0086841

**Published:** 2014-02-11

**Authors:** Melanie Oey, Ian L. Ross, Ben Hankamer

**Affiliations:** Institute for Molecular Bioscience, The University of Queensland, St Lucia, Queensland, Australia; University of South Florida College of Medicine, United States of America

## Abstract

With a rising world population, demand will increase for food, energy and high value products. Renewable production systems, including photosynthetic microalgal biotechnologies, can produce biomass for foods, fuels and chemical feedstocks and in parallel allow the production of high value protein products, including recombinant proteins. Such high value recombinant proteins offer important economic benefits during startup of industrial scale algal biomass and biofuel production systems, but the limited markets for individual recombinant proteins will require a high throughput pipeline for cloning and expression in microalgae, which is currently lacking, since genetic engineering of microalgae is currently complex and laborious. We have introduced the recombination based Gateway® system into the construction process of chloroplast transformation vectors for microalgae. This simplifies the vector construction and allows easy, fast and flexible vector design for the high efficiency protein production in microalgae, a key step in developing such expression pipelines.

## Introduction

By 2050, world population is projected to reach 9.6 billion and with continued economic development the demand for food and energy is forecast to increase by 70% (UN) and 50% (International Energy Agency), respectively. Microalgae technologies are positioned at the nexus of these challenges. Algae, as photosynthetic organisms, absorb solar energy and store it as chemical energy which is then usable for food and fuel. Furthermore microalgae can utilise non-arable land and employ non-potable water sources thereby contributing to a transition from a *food versus fuel* scenario to a *food and fuel* future.

In addition to fuel and biomass production, microalgae have been shown to be excellent candidates for cheap and scalable foreign protein expression. They therefore have the potential to form a rapid, scalable and economic production platform for high value proteins, for both animal and human food products, for industrially useful proteins such as enzymes and for ‘molecular pharming’ of therapeutic proteins, including vaccines and other therapeutic products [1]. Such high value co-products have the potential to subsidise biofuel production during industrial scale-up, making algal biotechnology a topic of broad interest.

### Algae as protein factories

The chloroplast is the preferred site for protein expression with expression levels up to 40% of total soluble protein [2], enabling protein folding, disulphide bonding [3], post-translational modification and the demonstrated ability to produce a variety of functional foreign proteins including vaccines, toxins and viral proteins [2]–[5]. The lack of epigenetic silencing also makes chloroplast transformants more stable and reliable [6] in comparison with genes inserted into the algal nucleus. The importance and potential of algal chloroplast protein production is highlighted by the recent production of antibody-linked toxins directed to cancer cells [3]. This was successful in algal chloroplasts and not in other established protein expression systems, due to the complex requirements for both prokaryotic (insensitivity to the toxin) and eukaryotic features (able to express functional antibodies).

### Chloroplast genome and foreign gene incorporation

The single cup-shaped chloroplast of *Chlamydomonas reinhardtii* carries around 50–100 genome copies (plastomes). The ∼200 kb circular plastome encodes around one hundred genes and shows a tetra partite structure, with two single subunits separated by two inverted repeats [7].

For high protein expression levels, a greater copy number of the introduced foreign DNA is beneficial [6]. Therefore introducing the foreign DNA into the inverted repeat region is useful as it theoretically allows doubling of the foreign DNA copy number (two foreign gene copies per plastome).

The incorporation of foreign genes into the chloroplast utilises homologous recombination. Efficient homologous recombination requires the insert to be flanked by sequences homologous to the target site of the chloroplast. This strategy ensures precise incorporation of the foreign DNA into the target site and prevents positional and epigenetic effects observed during nuclear transformation.

### Selection marker

Methods for the selection of successful clones vary from complementation of mutations (e.g. restoration of photoautotrophic growth) to the use of selectable marker genes conferring antibiotic or herbicide resistance [8]. The disadvantage of mutation complementation is the need for a mutant strain impaired in the flanking regions around the target insertion site. In contrast antibiotic resistance genes can easily be integrated with the inserted foreign genes.

### Gateway® site-specific recombination system

Traditional cloning of each new gene into a suitable antibiotic resistance vector is an obstacle to rapid strain development. The lambda-phage based Gateway® system overcomes this problem by making use of recombination sequences that facilitate the transfer of DNA fragments between vectors [9]. The lambda *att* recombination sequences, so called “att-sites”, flank both the sequence which is to be exchanged and the target site in the vector.

Using green fluorescent protein (GFP) as a demonstration system, we have utilised the Gateway® system to create a simple, flexible, easy to modify and interchangeable vector system that allows the rapid insertion of different expression cassettes into the destination vectors of choice. This supports the rapid development of *Chlamydomonas reinhardtii* protein overexpression strains suitable for high value product production. The widespread global use of the Gateway® system means that many genes of interest are readily available for rapid transfer in this manner.

## Materials and Methods

### Plasmid construction

#### Destination-vector

**Figure 1:**
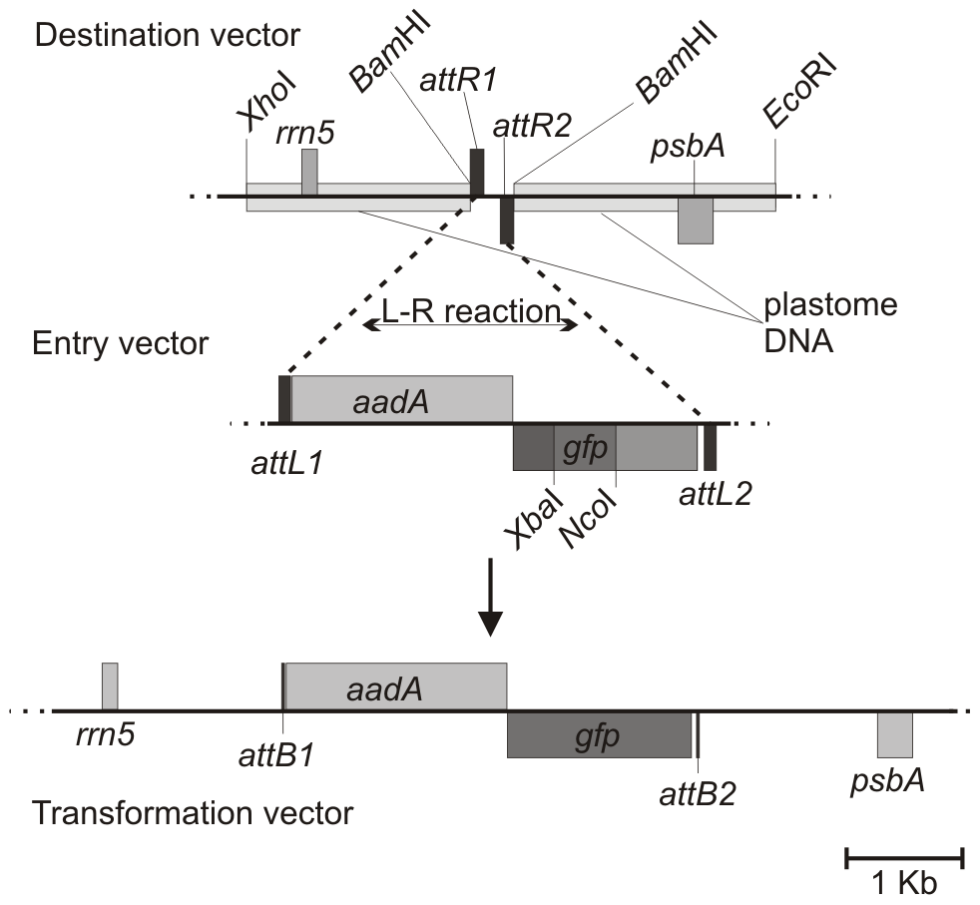
Schematic illustration of the chloroplast vector system. Destination vector and entry vector are recombined through L-R reaction yielding the final chloroplast transformation vector. Destination vector: Chloroplast homologous sequences (plastome DNA) with ribosomal (*rrn5*) and *psbA* (*psbA*) genes are indicated. *attR* sites (*aatR1*, *attR2*) supporting L-R reaction are indicated. Entry vector: *attL* sites (*attL1*, *attL2*) participating in L-R reaction are indicated as well as the expression cassette of the selection marker *aadA* and the *gfp* expression cassette. Important restriction sites used for cloning are indicated as well as the recombination reaction performed between the *attR* and *attL* sites (*L-R reaction*). Transformation vector: Schematic illustration of final transformation vector resulting from L-R reaction between destination and entry vector.

For the construction of the destination vector (Figure 1) the chloroplast homologous region of *P332* [10] was cloned as an *EcoR*I/*Xho*I fragment into the *pBluescript* derivative *pStore*. The Gateway® *attR1* and *attR2* sites with a linker of 156 bp were synthesised (sequence see Figure S1) and cloned into the *BamH*I site between the *psbA* and *rrn* operon. Cloning into a single insertion site resulted in different orientations of the *attR* sites versus the operons. Vectors with the *attR2* site oriented towards the *psbA* operon were called *pC-Dest/psbA* and vectors with the *attR2* site oriented towards the *rrn* operon were called *pC-Dest/rrn*.

The most rapidly synthesised protein at high light intensity in higher plants and algal cells is the D1 protein, the core protein of photosystem II, which is encoded by *psbA* [11]. The *pC-Dest/psbA* vector was therefore used as theoretically it should show higher expression levels due to read through from *psbA* compared to *rrn*.

#### Entry-vector

The GFP expression cassette was constructed by cloning *gfp* into *pStore* as an *Nco*I/*Xba*I fragment. The GFP gene was equipped with a *C. reinhardtii atpA* terminator (*TatpA*) which was amplified as a PCR product and cloned as an *Xba*I/*Xma*I fragment. Finally an *atpA* promoter-5′UTR (untranslated region) fragment from *pUC-atpX-aadA* [12] was cloned as a *Cla*I/*Nco*I fragment in front of the GFP to complete the expression cassette.

The selectable marker gene *aadA* was retrieved from *pUC-atpX-aadA*. To delete the *Nco*I cloning site, the *atpA* promoter (*PatpA*) was PCR amplified with Cr-PatpA-fwd (5′AAGCTTATCGATTGACTTTA3′) and Chloro-aadA-Pci-rev (5′AAAAAAACATGTACATTTTCACTTCTGGAGTGTATTG3′) and cloned as a *Cla*I/*Pci*I fragment into the *Cla*I/*Nco*I site. The final *aadA* gene was then PCR amplified with primers GATE-Chloro-aada-F (5′CACCGATTGACTTTATTAGAGGCAGTG3′) and GATE-Chloro-aada-R (5′AAGGTACCAACCCGGGTGGATCGCACTCTACCGATT 3′) and inserted into the *pENTR-D* topo vector using the *pENTR-D* topo cloning kit (Invitrogen, Invitrogen, Life Technologies Australia Pty Ltd, Mulgrave, Victoria, Australia). GATE-Chloro-aada-R is carrying a *Kpn*I and *Xma*I site which was used for subsequent introduction of the GFP expression cassette. To complete the entry vector (Figure 1), the GFP expression cassette was cloned tail-to-tail with the *aadA* selection marker using *Kpn*I/*Xma*I sites for insertion.

#### Algal strains, cultivation and transformation

Transformation was performed using *C. reinhardtii* strain *CC406* which was obtained from the Chlamydomonas Resource Center (http://chlamycollection.org/?s=cc406, accessed 19.11.2013). Algae were cultivated in TAP media [13] under continuous fluorescent white light (100–150 µE m^−2^ s^−1^, 1 µE = 1 µmol photons).

Chloroplast transformation was performed using biolistic bombardment [14]. In brief, *CC406* was grown to log phase (1–2×10^6^ cells/mL). 100 mL cell culture were centrifuged (10 min, 500×g, RT), the supernatant discarded and the cells were equally plated on three TAP agar plates containing 200 mg/L spectinomycin. 5 µg of vector were precipitated on 1.25 mg (sufficient for the bombardment of three different plates) of 0.6 µm gold particles (Bio-Rad Laboratories Pty Ltd, Gladesville, New South Wales, Australia) with the assistance of CaCl_2_ [2.5 M] and spermidine [0.1 M]. DNA coated gold particles were equally divided in three aliquots, pipetted on macrocarrier (Bio-Rad Laboratories Pty Ltd, Gladesville, New South Wales, Australia) and then fired onto the algae lawn using the PDS-1000/He™ system and rupture discs of 1100 psi (Bio-Rad Laboratories Pty Ltd, Gladesville, New South Wales, Australia). Plates were kept in low light (10 µE m^2^ s^1^) until colonies appeared. Colonies were picked and cultivated in liquid media supplemented with 200 mg/L spectinomycin and used for subsequent experiments.

### Mutant identification and protein extraction

To identify GFP expressing mutants, 50 mL of cell culture was centrifuged (10 min, 500×g, RT) and the supernatant discarded. The cell pellet (∼100 µL) was then resuspended in 500 µL protein extraction buffer (HEPES-KOH, pH 7.5 [50 mM], potassium acetate [10 mM], magnesium acetate [5 mM], EDTA [1 mM], 1× Complete™ Protease Inhibitor – EDTA free (Roche Australia, Brisbane, Queensland, Australia)) with DTT [1 mM final concentration] freshly added. Around 100 µL of dry 425–600 µm acid washed glass beads (Sigma-Aldrich, Castle Hill, New South Wales, Australia) were added to the cell culture to enhance cell rupture. Three cycles of freezing the sample in liquid N_2_, thawing and vortexing at maximum speed were conducted before the sample was centrifuged at 12000×g for 10 min at 4°C. The supernatant was taken to estimate the total soluble protein, determined using a Nanodrop 2000c spectrophotometer (Thermo Fisher Scientific Australia, Brendale, Queensland, Australia). Native PAGE was conducted (Separation gel buffer: 1.5 M Tris-HCl pH 8.8; stacking gel buffer; 0.5 M Tris-HCl pH 6.8; running buffer; 0.03 M Tris-HCl pH 8.3, 0.2 M Glycin; 15% acrylamide) and GFP intrinsic fluorescence was detected using a ChemiDoc™ MP Imaging system (Bio-Rad Laboratories Pty Ltd, Gladesville, New South Wales, Australia) to demonstrate the presence of GFP protein. The control GFP lane utilised recombinant GFP protein which was kindly provided by Fabian Kurth (Institute for Molecular Bioscience, University of Queensland).

## Results and Discussion

To harness microalgae for the efficient foreign protein production, chloroplast transformation is the method of choice, providing high expression levels. Successful and simple chloroplast transformation depends on the construction of an appropriate transformation vector and multiple factors must be considered such as final foreign DNA copy number in the plastome and on-site location of the selection marker gene. However, with each additional feature, the plasmid size increases and becomes more difficult to handle, requiring a simple, easy and streamlined construction method.

To increase the final foreign DNA copy number, the intergenic region in the inverted repeats between *psbA* and *rrn* operon of the *C. reinhardtii* plastome are well suited as target site as it theoretically doubles the gene copy number. However intergenic regions are poorly conserved, show a high degree of repetitive sequences and differ even between laboratory strains of the same species [7]. Consequently, conserved flanking genes should be incorporated into the transformation vector to secure precise insertion. For *C. reinhardtii* the intergenic region in the inverted repeats including the flanking *rrn5* gene and the 3′ exon of the *psbA* gene is about 3.5 kb. When inserted in a standard *pBluescript* vector it yields a basic homologous recombination vector of ∼6.5 kb in size.

**Figure 2:**
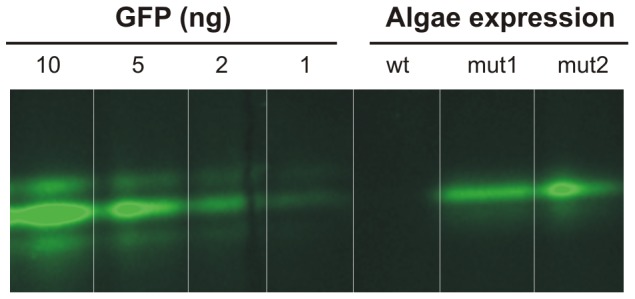
GFP fluorescence detection via Native PAGE. To identify GFP expressing mutants 1(wt), mutant 1 (mut1) and mutant 2 (mut2) algae were loaded and intrinsic GFP fluorescence was used for detection. 10, 5, 2 and 1 ng of GFP protein were used as standard.

Although co-transformation of a plasmid carrying the DNA of interest and a plasmid carrying a marker gene has been reported [10], this strategy often results in low levels of mutant recovery [15]. Therefore, the insertion of a ∼2 kb selectable marker gene alongside the ∼2 kb GFP expression cassette (as described in this paper) between the homologous recombination regions has the advantage of using the marker gene as on-site confirmation of the right location and functionality within the chloroplast genome. However, this increases the size of the vector to >10 kb. The intended insertion of several genes of interest organised into an operon-like structure into the chloroplast transformation vector would result in an unmanageable plasmid size, a major obstacle to success. Large vectors are generally difficult to handle when used for traditional cloning operations utilising restriction enzymes and ligase and therefore create difficulties for rapid cloning, making this unsuitable as a generic high-throughput approach. We have overcome this problem by creating a recombination-based vector set using the Gateway® system as starting point. In this set, a so called “entry vector” was created by inserting the marker gene and a tail-to-tail oriented GFP expression cassette into the 2.5 kb *pENTR-D* topo vector. This led to both cassettes being flanked by *attL* sites (Figure 1) and in parallel yielding a comfortably sized (∼6 kb) working plasmid for simple modifications and fragment exchanges. For the so called “destination vector” we cloned the chloroplast target sequence into a *pBluescript* vector and inserted *attR* sites in the intergenic region (Figure 1). The organization of the gene of interest and the chloroplast target sequence on two different plasmids has the advantage of allowing flexible interchange between them. Consequently one entry vector can serve several destination vectors, which would allow, for example, expression levels at different locations of the chloroplast genome to be compared using the same expression cassette. Conversely, one destination vector can be used in combination with several entry vectors supporting rapid development of new vectors for the expression of foreign proteins in algal strains. In the present example, an L-R recombination reaction [9] between both destination and entry vector resulted in precise introduction of the expression cassettes of marker and GFP gene in the inverted repeat region, creating the final vector which was used for transformation. The transformation generated >150 colonies per plate corresponding to a transformation efficiency of ∼100 transformants/µg DNA. We selected several putative positive mutants displaying spectinomycin resistance and applied native PAGE to successfully detect GFP expression in extracted total soluble protein. Several transformants were shown to produce GFP of which two examples are shown in Figure 2. Bacterially expressed recombinant GFP was used as a protein standard. As expected, the initial expression level was low (∼5 µg/g of total soluble protein) as the chloroplast genome is not currently homoplastic and a codon optimised *gfp* version (∼80-fold increase in expression level [10]) was not used for these proof of principle experiments. These additional modifications are expected to greatly improve expression levels towards the current published maximum of 40% of total soluble protein [2], [10], [16].

Therefore, it is likely that both screening and protein expression levels can be improved further by incorporating those aspects. At present, screening of the recombined plasmids is required to select a correct clone for transformation. By including the commonly used *ccdB* toxin gene between the *attR* sites of the destination vector (which would be eliminated during recombination) would allow growth only of *E. coli* colonies carrying the correctly assembled final vector [17]. The *ccdB* gene with 2.5 kb was deliberately not included here to limit the size of the destination vector during proof of concept. Having achieved this, the insertion of the *ccdB* gene to streamline cloning procedures will be explored.

## Conclusion

We have employed the Gateway® system to simplify and streamline the cloning steps towards a transformation vector designed for the microalgae *Chlamydomonas reinhardtii* chloroplast. The development of this tool will accelerate the development of microalgae cell lines for targeted recombinant protein expression and thus enable the rapid, flexible and high capacity production from these algal systems.

## Supporting Information

Figure S1 **Sequence of synthesized** ***attR*** **fragment.**
(DOC)
