# Supplementary material for: Gateway-Assisted Vector Construction to Facilitate Expression of Foreign Proteins in the Chloroplast of Single Celled Algae
Source: PLoS One. 2014 Feb 11;9(2):e86841. doi: 10.1371/journal.pone.0086841 (PMC3921121; doi:10.1371/journal.pone.0086841)
Supplement: Figure S1 — Sequence of synthesized attR fragment. (DOC) [file pone.0086841.s001.doc]

Supplementary data:

Sequence of synthesized *attR1/2* sites:

gcatggatacggatccacaagtttgtacaaaaaagctgaacgagaaacgtaaaatgatataaatatcaatatattaaattagattttgcataaaaaacagactacataatactgtaaaacacaacatatccagtcactatgtctagagcggccgctaagttggcagcatcacccgacgcactttgcgccgaataaatacctgtgacggaagatcacttcgcagaataaataaatcctggtgtccctgttgataccggatgtcaggctcccttatacacagccagtctgcaggtcgaccatagtgactggatatgttgtgttttacagtattatgtagtctgttttttatgcaaaatctaatttaatatattgatatttatatcattttacgtttctcgttcagctttcttgtacaaagtggtggatccagcgatatta

attR1

attR2
